# Addition of Epidermal Growth Factor Improves the Rate of Sulfur Mustard Wound Healing in an In Vitro Model

**Published:** 2008-03-26

**Authors:** Claudia L. Henemyre-Harris, Angela L. Adkins, Augustine H. Chuang, John S. Graham

**Affiliations:** Physiology and Immunology Branch, Research Division, US Army Medical Research Institute of Chemical Defense, Aberdeen Proving Ground, MD; Department of Clinical Investigation, Dwight D. Eisenhower Army Medical Center, Fort Gordon, GA; Medical Toxicology Branch, Analytical Toxicology Division, US Army Medical Research Institute of Chemical Defense, Aberdeen Proving Ground, MD

## Abstract

**Objective:** Sulfur mustard (SM) causes blisters on the human skin. These blisters delay healing of the skin and make the victims more susceptible to infection. In vitro models have been used for protection studies against SM injury, but study on wound healing after SM exposure has not been explored. The purpose of this study was to test whether the addition of exogenous growth factors could improve the rate of SM wound healing. **Methods:** The model consisted of normal human epidermal keratinocytes seeded into 6-well plates, exposed to SM, and wounded (disruption of the cell monolayer) with a sterile wounding instrument. Cells were then stained and images were captured to measure percentage wound fill. Epidermal growth factor (EGF) and keratinocyte growth factor (KGF) were tested in this model. **Results:** EGF (1 ng/mL) significantly increased wound fill on all of the days tested (days 6, 9, and 12). KGF did not significantly improve wound healing. **Conclusions:** EGF showed promise as a potential therapy for SM-induced wounds. This in vitro model was a valuable tool for screening therapeutics before animal testing. These results will be used to develop a dressing that can slowly release EGF on to a debrided wound bed to help speed the healing process.

## WOUND HEALING OVERVIEW

An understanding of normal wound healing is imperative for the development of an appropriate in vitro model for sulfur mustard (SM) injury. However, the topic of wound healing is too complicated to be covered in its entirety in this article. Therefore, this article addresses only some of the key points of the predominant phases of wound healing to provide a brief overview of this complex topic. Skin contains 2 main tissue layers, an epidermal layer of stratified squamous epithelium consisting of 5 different sublayers and an underlying dermal layer of connective tissue rich in collagen. Hair and glands are derived from the epidermis but protrude deep into the dermal layer.^1^

Wound healing is a continuous and complex process that is generally characterized as having 4 overlapping phases: coagulation, inflammation, proliferation, and remodeling.^2^ Coagulation begins immediately following injury. A clot consisting of platelets and fibrin fibers is formed to maintain hemostasis, shield the wounded tissue, and serve as a scaffold for cells migrating into the wound. The clot also serves as a reservoir of growth factors and cytokines that attract inflammatory cells, such as neutrophils and monocytes, to the wound site.^1^ Inflammation begins within minutes after the injury. Neutrophils clear out contaminating bacteria and release cytokines to activate fibroblasts and keratinocytes. Macrophages phagocytize any remaining pathogens, debride the wound, and secrete growth factors and cytokines that stimulate fibroblasts and endothelial cells.^2^ During the proliferative phase, endothelial cells, fibroblasts, and keratinocytes proliferate and migrate into the wound to repair vascular damage, replace destroyed tissue, and resurface the wound. Endothelial cells detach from undamaged blood vessels via collagenase, migrate into the wound, and proliferate to restore vascular integrity.^3^ Dermal fibroblasts adjacent to the wound site proliferate early after injury, and then around 3 or 4 days after injury, migrate into the wound clot and lay down a matrix rich in collagen.^1^

Within hours after injury, keratinocytes begin to migrate into the wound. After several days, migrating keratinocytes proliferate, form a protective monolayer over the wound, and reestablish contact with the underlying basement membrane in a process called *epithelialization*.^3^ This process is aided by contraction of the underlying connective tissue, which brings the edges of the wound closer together. The keratinocytes then cease to proliferate and migrate, but instead differentiate to form a new stratified epidermis attached to a basal lamina.^2^ Proteases play an important role in epithelialization. They are thought to be responsible for releasing keratinocytes from the basal lamina and for permitting leading-edge keratinocytes to pass through and along the fibrin clot.^1^ During the remodeling phase of wound healing, proteases remove excess collagen and matrix. Collagen fibers increase in thickness and realign to give strength to the healing tissue. Remodeling may take up to a year to complete, but will never result in tissue as strong as the original uninjured tissue.^2^

## THE SM THREAT

Mustard was first used in World War I and was responsible for an estimated 80% of all chemical casualties.^4^ Recent terrorist attacks and the use of SM in military conflicts such as the Iran-Iraq War of the 1980s have stressed the need for the development of pharmaceutical treatments for the clinical management of chemical warfare agent casualties. Current treatments for SM injuries include deroofing, debridement, irrigation, topical antibiotics, and sterile dressings.^4^,^5^ However, these treatments need to be improved to eliminate infections, enhance cosmetic outcomes, and improve wound healing. Graham et al^5^ have comprehensively reviewed strategies for developing improved therapies for cutaneous SM injuries. Appropriate in vitro screening techniques, before animal testing, may speed up the development of clinical treatments.

Mustard is a persistent, oily chemical agent that can cause injury to the eye, airway, and skin. Initial clinical symptoms in the skin include erythema followed by vesication, the extent of which is dependent on the level of exposure to SM. SM (2,2′-dichlorodiethyl sulfide) reacts with nucleophilic sites such as DNA, proteins, and membrane components, and several mechanisms for damage have been suggested.^6^ The basal epidermal cell is a primary target for SM.^6^,^7^ Ultrastructural studies using transmission and scanning electron microscopy were performed on SM-treated hairless guinea pig skin and cultured human keratinocytes. These studies revealed that the nucleus, plasma membrane, and anchoring filaments of the epidermal basal cells were damaged. This damage led to separation of the epidermis from the underlying dermis and production of a subepidermal microblister in the skin of the hairless guinea pig.^4^,^7^

## IN VIVO CUTANEOUS WOUND HEALING MODELS FOR SM

Appropriate in vivo and in vitro models are critical for the development of clinical treatments. These models are especially important for developing SM therapies because human clinical trials cannot be conducted for ethical reasons. A number of animal models have been used to study SM-induced cutaneous lesions, including the hairless guinea pig, mouse ear, hairless mouse, and the weanling pig. Although no one animal model is perfect for studying all aspects of human blister formation, these models have proven valuable for studying various aspects of SM injury.^8^ In general, the mouse ear seems to be the most resource-efficient animal model for initial drug screening. Pig skin is most similar to human skin and appears to be the best animal model for testing drugs, studying histopathology, and developing wound healing therapies.^5^,^8^ In particular, weanling pigs have been used extensively as an animal model for efficacy testing of candidate treatment regimens.^5^,^9^,^10^ They were used to test laser debridement,^11^ sterile dressings, and allograft materials.^12^ In addition, several noninvasive bioengineering techniques were developed to determine lesion depth^13^,^14^ and to monitor wound healing progress following SM exposure.^15^

However, animal models can be time consuming and costly. An in vitro wound healing model could be used as a first-line screen before animal testing, potentially minimizing cost, reducing animal use, and expediting fielding of wound healing products. Complementary in vitro and in vivo studies could provide stronger evidence than in vivo studies alone that a candidate therapy should be used to treat an SM injury.

## IN VITRO CUTANEOUS WOUND HEALING MODELS FOR SM

Current in vitro cutaneous models focus on understanding the biochemical mechanisms of SM-induced cutaneous injury with the ultimate goal of developing medical countermeasures to prevent blister formation. Human epidermal keratinocytes are widely used to model the cytotoxic effects of SM on basal epidermal cells^16^ and have been used to study a number of cellular processes, including protease induction,^17^ apoptosis,^18^ and metabolism.^19^ Primary rat keratinocyte cultures^20^ and a number of immortalized human skin cell lines, including G361, SVK14, HaCaT, and NCTC 2544, have also been used to study SM toxicity.^21^ Organ culture of full-thickness human skin explants exposed to SM were used to identify mediators of the inflammatory response.^22^ Another research team used bioengineered human skin grafted to nude mice to study the early events in SM-induced cutaneous injury. This bioengineered skin demonstrated many of the alterations found in animal models of cutaneous SM injury, but the latest time point in which it was evaluated was 24 hours.^23^

Medical prophylactics are critical to protecting soldiers and civilians from an SM attack. However, not everyone in the attack will be fully medically prepared to prevent SM injury. There will be victims even if the prophylactic is considered 100% medically effective because, in reality, consumer use errors will make the prophylactic less than 100% effective. One in vitro model was developed to investigate the mechanisms involved in delayed healing of SM wounds. This model consisted of a 3-dimensional culture of human fibroblasts in collagen gel surrounded by normal human keratinocytes and elucidated some of the dermal-epidermal interactions in normal skin.^24^ However, this model was not used to test therapies to combat delayed healing of SM wounds.

The purpose of this study was to test whether the addition of extrinsic growth factors could help improve the rate of SM wound healing. Epidermal growth factor (EGF) and keratinocyte growth factor (KGF) were evaluated in an in vitro wound healing model.

## MATERIALS AND METHODS

### Cell cultures

Normal human epidermal keratinocytes (NHEKs) were obtained from Dr William Smith's laboratory at the US Army Medical Research Institute of Chemical Defense from commercial sources (Cambrex Bioscience, Walkersville, Md), as previously described.^25^ Cells (third passage) were seeded into 6-well plates (Corning Corporation, Corning, NY) and grown to 30 to 40% confluency in keratinocyte growth medium (KGM, Cambrex Bioscience).

### SM wound production

Cells were exposed to 0, 5, 10, 25, 50, or 100 μM of SM, in KGM, in a chemical surety hood for 1 hour.^16^ Cells were transferred to an incubator for an additional 2 hours of SM exposure at 37°C and 5% CO_2_ and then used in the wound healing model. A 3-mm wide wound (disruption of the cell monolayer) was created with a sterile wounding instrument.^26^,^27^ Cells were rinsed, and the wounded area was examined microscopically to ensure that cellular debris was removed. Wounds were marked on the bottom of the plate with a blue, ultrafine point permanent marker (Sharpie, Sanford Corporation, Oak Brook, Ill), and the wells received fresh media (with or without growth factor treatment).

### Growth factor treatments

EGF (#1376454, Roche Molecular Biochemicals, Indianapolis, Ind) was resuspended in sterile H_2_O and diluted to its final concentrations (1, 10, or 20 ng/mL) in KGM. KGF (recombinant human [rh] KGF, #N000173, Amgen, Inc., Thousand Oaks, Calif) was resuspended in sterile H_2_O, diluted in PBS containing bovine serum albumin (0.1%) to prevent degradation, and diluted to its final concentrations (10, 50, or 100 ng/mL) in KGM. For both growth factors, half volume media changes (with fresh growth factor) were performed daily. Control NHEKs received daily half volume medium changes with KGM alone. Half volume media changes were conducted to provide fresh growth factor to cells and to reduce disruption of the cellular environment within the well in the event that cells secreted autocrine factors.

### Crystal violet stain

Cells were stained with the cytoplasmic stain crystal violet (#C3886, Sigma-Aldrich, St Louis, Mo), as previously described.^28^ Briefly, cells were fixed with 4% paraformaldehyde, rinsed 3 times with 0.1 M of phosphate buffer, and stained for 1 minute in 0.1% crystal violet (wt/vol) in deionized water. Cells were washed with deionized water and were air dried.

### Wound fill measurements

Images of stained cells were captured using a digital camera (Coolpix, Nikon Instrument Inc., Lewisville, Tex) and a dissecting microscope (#SZX12, Olympus, Center Valley, Pa). Images were then analyzed with Image-Pro Plus 5.0 software (Media Cybernetics, Bethesda, Md) to determine percentage wound fill. Each image was analyzed 3 times.

### Statistical analyses

Data are reported as mean ± standard error of the mean (SEM). Statistical significance was defined as *P* ≤ .05 for all tests. Analyses were conducted using Statistical Analysis System software (SAS Institute, Inc., Cary, NC). Depending on the number of variables involved in a particular study (ie, SM concentration, drug treatment, day cells stained), a 2-sample *t* test or an analysis of variance was conducted. A Tukey test was then used to compare the pairs of treatment groups. See figure legends for the specific statistical test conducted.

## RESULTS

### Test of EGF

**Figure 1:**
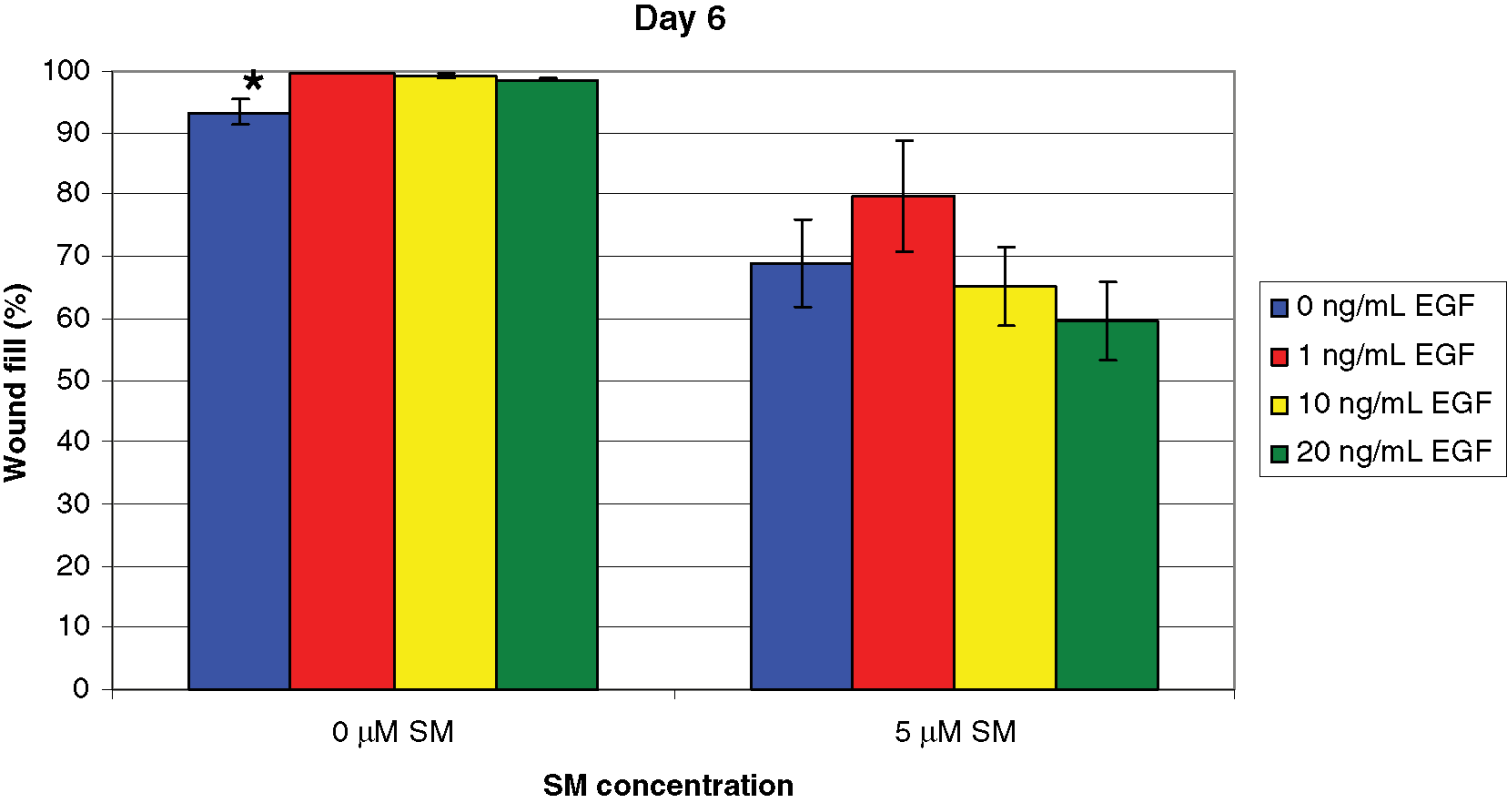
NHEK cells exposed to 5 μM of SM and treated with EGF. Cells were treated daily with 0, 1, 10, or 20 ng/mL of EGF and stained with 0.1% crystal violet 6 days after wounding. For 0 μM of SM, a significant difference* (*P* ≤ .05) in wound fill was observed between the cells treated with 0 ng/mL of EGF and the 1, 10, and 20 ng/mL of EGF groups. For 5 μM of SM, cells treated with 1 ng/mL of EGF had the greatest percentage of wound fill, but no statistically significant differences were observed between the EGF treatment groups. Data points represent mean values ± SEM of 3 determinations. A 1-factor ANOVA was used to compare treatment groups at each SM concentration.

EGF was tested in the in vitro wound healing model. In the first study, NHEK cells were exposed to 5 μM of SM and treated daily with KGM containing 0, 1, 10, or 20 ng/mL of EGF (Fig 1). For the cells exposed to 0 μM of SM, there was a 6% increase in wound fill in cells that received EGF versus those NHEKs that did not (*P* ≤ .05). For cells exposed to 5 μM of SM, data suggested that the cells treated with 1 ng/mL of EGF had the highest percentage wound fill, but this difference was not found to be statistically significant (*P* ≤ .05).

**Figure 2:**
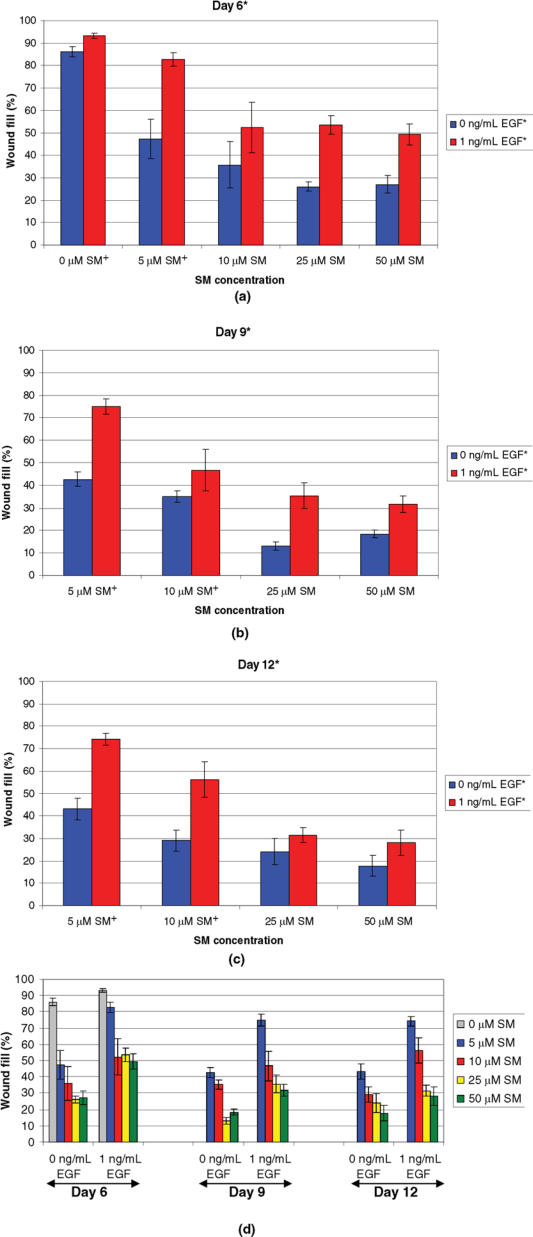
Wound fill time-course study for NHEK cells exposed to various concentrations of SM and treated with 1 ng/mL of EGF. Cells were exposed to 0, 5, 10, 25, or 50 μM of SM, treated daily with 0 or 1 ng/mL of EGF, and stained with 0.1% crystal violet at 6 (a), 9 (b), or 12 (c) days after wounding. A significant difference* was observed between the 2 treatment groups, 0 and 1 ng/mL of EGF, on days 6, 9, and 12. However, no significant interactions were observed between SM concentrations and EGF doses, so it cannot be specifically stated that there was a significant difference between EGF doses at a particular SM concentration (ie, 0, 5, 10, 25, or 50 μM of SM). Significant differences between SM concentrations,† regardless of EGF dose, were observed at each day. (a) For day 6, 0 μM of SM† had significantly different wound fill than 5, 10, 25, and 50 μM of SM. In addition, 5 μM of SM† had significantly different wound fill than 10, 25, and 50 μM of SM. (b and c) For days 9 and 12, 5 μM of SM† had significantly different wound fill than 10, 25, and 50 μM of SM and 10 μM of SM† had significantly different wound fill than 25 and 50 μM of SM. Data points represent mean values ± SEM of 6 determinations from 2 separate experiments. A 2-factor ANOVA at each staining day was used to compare the SM concentrations and the 2 EGF doses. Statistical significance was defined as *P* ≤ .05 for all tests. (d) Summary graph of parts (a)–(c).

In a second study, the most promising EGF concentration (1 ng/mL) was tested after exposure to 0, 5, 10, 25, or 50 μM of SM (Fig 2). Regardless of the SM concentration tested, NHEK cells treated with 1 ng/mL of EGF had significantly more wound fill than those cells that received cell culture medium without 1 ng/mL of EGF, at all days tested (days 6, 9, and 12).

### Test of KGF

**Figure 3:**
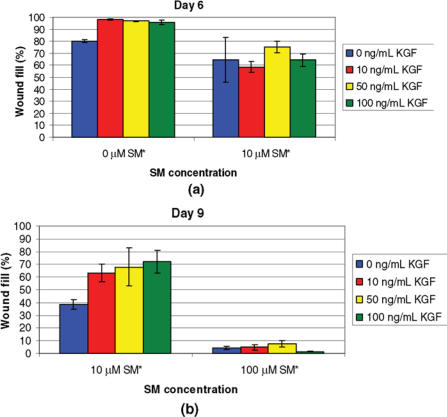
Wound fill time-course study for NHEK cells exposed to various concentrations of SM and treated with 0, 10, 50, or 100 ng/mL of KGF. Cells were exposed to 0, 10, or 100 μM of SM, treated daily with the appropriate KGF concentration, and stained with 0.1% crystal violet at 6 (a) or 9 (b) days after wounding. (a) A significant difference* in percentage wound fill was observed between 0 and 10 μM of SM on day 6. No significant differences between KGF doses were observed for day 6. (b) A significant difference* in wound fill was observed between 10 and 100 μM of SM on day 9, but no significant differences were noted between the KGF doses. Data points represent mean values ± SEM of 3 determinations. A 2-factor ANOVA was used to compare the treatment groups and SM concentrations at each day, followed by a Tukey post hoc comparison of pairs of treatment groups. Statistical significance was defined as *P* ≤ .05 for all tests.

KGF was tested in the in vitro wound healing model. NHEK cells were exposed to 0, 10, or 100 μM of SM and treated daily with KGM containing 0, 10, 50, or 100 ng/mL of KGF (Fig 3). A significant difference in percentage wound fill was observed between 0 and 10 μM of SM on day 6, regardless of the KGF concentration tested. The cells exposed with 10 μM of SM had 15% to 32% less wound fill than cells not exposed to SM. No significant differences between KGF doses were observed for day 6. A significant difference in percentage wound fill was also observed between 10 and 100 μM of SM exposed cells on day 9. The cells exposed with 100 μM of SM had 34% to 71% less wound fill than those exposed with 10 μM of SM. The data suggest that following exposure to 10 μM of SM, the addition of KGF at any concentration tested improved wound healing compared with untreated control cells. However, no significant differences between KGF concentrations were noted for day 9 (*P* ≤ .05).

## DISCUSSION

The purpose of this study was to determine whether the addition of extrinsic growth factors could improve the rate of wound healing following SM exposure. NHEKs were chosen because they appear to be a good model for basal epidermal cells,^29^ a principal target for SM-induced skin lesions.^6^,^30^ The 3-hour SM exposure time (1 hour in the hood and 2 hours in the incubator) was chosen to make this model as clinically relevant as possible and to meet laboratory safety standards for working with SM. Laboratory specimens must remain in a chemical fume hood for a minimum of 1 hour after SM exposure to off-gas, per safety regulations. After the initial 1 hour, cells are placed in an incubator at 37°C for an additional 2 hours for a total SM exposure of 3 hours. Erythema begins 1 to 24 hours post-SM exposure and is characteristically seen 4 to 8 hours after SM exposure.^4^ Many victims do not realize they were exposed to SM and do not take corrective action until erythema appears. The 3-hour SM exposure time for the in vitro model correlates with what is seen clinically.

The SM concentrations selected for these experiments were based on a previous study. A liquid droplet of about 10 μg of SM produces vesication on the skin.^4^ Previous studies determined lethal and sublethal concentrations of SM on cultured HEKs.^16^,^19^ The SM concentrations used in this study were selected on the basis of personal communication with Dr Bill Smith of US Army Medical Research Institute of Chemical Defense, as described previously. This study showed that SM demonstrated a concentration-dependent decrease in wound fill by NHEK cells as the SM concentration increased. In general, regardless of SM exposure, wound fill increased from day 3 to day 6, reached its highest values at day 6, remained constant or slightly decreased at day 9, and finally decreased at day 12.^31^

The daily treatment regimen was selected on the basis of the frequency with which one might change a dressing on a small wound. Autocrine factors could easily be removed in a cell culture model if a complete media change was performed daily. For this reason, half volume media changes were conducted to provide fresh growth factors to cells and to reduce disruption of the cellular environment within the well.

A candidate growth factor for the in vitro wound healing model is EGF (*β*-urogastrone). EGF is expressed in cells by various adult tissues and its production is increased by testosterone and decreased by estrogen.^32^ EGF was shown to increase the motility of keratinocytes and many other cell types.^33^ Keratinocyte colonies treated with EGF had an 8-fold greater rate of increase of colony radius than untreated control colonies. The colony growth effect of EGF was reported to be because of EGF's ability to increase the rate of cell migration in the cells located along the peripheral border of the colony.^34^ EGF induced migration and contraction of primary cultures of human keratinocytes and stimulated the mitogen-activated protein kinase signal transduction pathway, a requirement for reepithelialization.^35^

In normal human skin, high concentrations of EGF receptor were found on basal keratinocytes.^33^ EGF receptors are measured clinically (testing available from several reference laboratories such as Quest Diagnostics and Mayo Medical Laboratories), but serum EGF is not routinely measured clinically (Dr Joseph Wood, PhD, MD, Endocrine, Diabetes, and Metabolism Service, Dwight D. Eisenhower Army Medical Center, Fort Gordon, Ga, written communication, 2007). One research study did evaluate EGF serum levels by enzyme immunoassay in schizophrenic and control patients. The serum EGF concentration was measured at 554 ± 350 pg/mL in the 14 control patients tested.^36^ In comparison, this study tested 1000, 10,000, and 20,000 pg/mL of exogenously added EGF (concentrations were based on manufacturer's recommendations for serum-free cell culture systems).

In this study, NHEK cells treated with 1 ng/mL of EGF following SM exposure had significantly greater wound fill than untreated, SM-exposed NHEK cells. This finding is consistent with other in vitro, in vivo, and clinical studies. Best growth of oropharyngeal keratinocytes was achieved with an EGF concentration of 0.15–1.5 ng/mL.^37^ In animal studies, topical application of biosynthetic EGF accelerated epidermal regeneration in split-thickness wounds in the pig.^38^ A rat burn study demonstrated that EGF administration accelerated healing of the burn wound on the skin.^39^ Topical application of rhEGF ointment promoted wound healing by myofibroblast proliferation and collagen synthesis in the rat.^40^ In humans, a phase III clinical trial conducted in India demonstrated the safety and efficacy of using an rhEGF gel (REGEN-D 150, Bharat Biotechs International Limited, Hyderabad, India, 150 mg/g) to treat diabetic foot ulcers.^41^ A phase IV, postmarketing surveillance study of REGEN-D 150 confirmed faster healing of diabetic foot ulcers and an increase in percentage of patients cured, and recorded no adverse events in patients enrolled in the study.^42^

KGF/fibroblast growth factor 7 is another candidate for clinical applications involving proliferation and stimulation of epithelial cells.^43^ KGF promoted cell motility in an in vitro wound healing model of alveolar epithelium.^44^ In addition, normal human keratinocytes treated with a combination of KGF and the predominant glycosaminoglycan in skin, dermatan sulfate, were stimulated to grow in culture.^45^ Human KGF topically applied to porcine models of partial- and full-thickness wounds stimulated the rate of reepithelialization.^46^ A World Health Organization reference standard for KGF was recently established in 2006,^47^ but routine KGF clinical testing is not currently available (Dr Joseph Wood, PhD, MD, Endocrine, Diabetes, and Metabolism Service, Dwight D. Eisenhower Army Medical Center, Fort Gordon, Ga, written communication, 2007).

In vitro, KGF was tested at concentrations of 1, 10, and 100 ng/mL in bovine corneal wound cultures over a 5-day period. At day 2 after wounding, bovine corneal wounds treated with 100 ng/mL of KGF had significantly greater reepithelialization than untreated control corneas. However, lower doses of KGF had no effect, nor did the 100 ng/mL of KGF dose, after the day 2 time point. This study also demonstrated that 1 ng/mL of KGF, but not 10 and 100 ng/mL of KGF, stimulated bovine keratinocyte migration when evaluated 8 hours after wounding. Later time points were not included in this study.^48^ This study of human keratinocytes tested 0–100 ng/mL of KGF at days 6 and 9 after wounding and reported no significant improvement in wound fill. These data are consistent with the later time points of the bovine corneal wound experiments. Perhaps, if KGF testing had been done at earlier time points for this study, KGF-stimulated wound fill improvement might have been detected in the human keratinocyte wound-healing model.

Clinically, KGF is a promising mitogen. Human recombinant KGF, commercially available as palifermin (Kepivance, Amgen, Inc.), received US Food and Drug Administration approval for the treatment of severe oral mucositis in patients receiving intensive chemotherapy followed by stem cell transplant for hematologic cancers.^49^ Palifermin was shown to reduce both the duration (6 days vs 9 days, *P* < .001) and incidence (63% vs 98%, *P* < .001) of grade 3 or 4 oral mucositis as compared with the placebo group.^50^ Palifermin has also had success in phase I and II clinical trials for treating mucositis in patients with solid tumors who receive chemotherapy with or without radiation therapy.^49^ The Mucositis Study Group of the Multinational Association of Supportive Care in Cancer and the International Society for Oral Oncology updated their clinical practice guidelines for the prevention and treatment of mucositis.^51^ One change from the original 2004 guidelines^52^ is the recommendation for the use of palifermin for oral mucositis associated with stem cell transplantation. The panel recommends a dose of 60 μg/kg of palifermin per day for 3 days before conditioning treatment and 3 days after transplantation to prevent oral mucositis.^51^ This study of human keratinocytes did not test KGF as a prophylaxis to SM-induced injury, but this concept will be incorporated into future studies.

One limitation of this study is that it addresses only the effects of SM on 1 cell type involved in the wound healing process. Keratinocytes may be a principal target for SM-induced skin lesions,^6^,^30^ but other cell types are destroyed in chronic SM wounds. Migrating and proliferating cell types involved in wound healing, such as fibroblasts and endothelial cells, are also good candidates for SM in vitro studies. A bioengineered human skin model is currently being used to identify early events in SM-induced skin injuries.^23^ Further development of this model could lead to a human skin model for studying wounds created by SM.

Another limitation of this study is that it does not address the issue of proteases. In a study of chronic and acute wound fluids, a 30-fold elevation of matrix metalloproteinase activity was observed in chronic wounds in comparison with acute wounds. There was also significantly higher degradation of exogenously added EGF in chronic wounds than acute wounds.^53^ SM stimulated an approximately 80-KDa serine protease release in cultured NHEKs. Protease activity at the dermal-epidermal junction is thought to lead to skin degradation, fluid accumulation, and blister formation.^17^ Exogenous growth factors added to this protease-rich environment could be destroyed before they could improve wound healing. Adding protease inhibitors, along with growth factors, to a wound could indirectly protect the growth factors long enough to improve wound healing. In fact, guinea pigs pretreated with the protease inhibitor doxycycline before SM intratracheal intoxication displayed less inflammation and histological epithelial lesions.^54^ SM-induced protease activity could destroy exogenously added growth factors before they could improve wound healing. Cell-controlled delivery of KGF via a fibrin gel improved wound healing both in an in vitro model of lung epithelium and in an in vivo full-thickness wound model (hybrid of human bioengineered skin transplanted on to athymic mice) as compared with the same models that received topically applied KGF.^55^ Protection and proper delivery are imperative for the growth factor to exert its effect. For these reasons, future experiments will be conducted with a concomitant use of antiproteases and growth factors in various delivery modes.

In conclusion, EGF showed promise in improving healing of SM skin injuries. KGF may exert its effects earlier in the wound healing process. This in vitro wound healing model was a useful tool for evaluating therapeutics for cutaneous SM injuries before animal testing. The information gained from this study will be used to develop a dressing that can slowly release, among other things, EGF on to a debrided wound to help speed the healing process. Protease inhibitors will also be considered in the development and/or application of this dressing.

## ACKNOWLEDGMENTS

The authors thank Drs Bill Smith and Margaret Martens for scientific guidance and Theresa Nipwoda and Eric Nealley for the NHEK cell preparations. We also thank Kristen Newkirk, Shuqunta Davis, and Tracey Hamilton for technical assistance, Charlene Corun, Juanita Guzman, and Marian Nelson for SM exposures, and Robyn Lee for statistical support. This project would not have been possible without the generous start-up funds from US Army Medical Research Institute of Chemical Defense commanders Colonel James Romano and Colonel Gennady Platoff. This project was funded by the Defense Threat Reduction Agency, Cutaneous Therapeutics Task Area (grant #L0007_04_RC_C) and was presented in part at the Medical Defense Bioscience Review, Hunt Valley, Md, May 2004, and at the 19th Annual Clinical Symposium on Advances in Skin & Wound Care, Phoenix, Ariz, 2004. Keratinocyte growth factor was generously provided via a Material Transfer Agreement with Amgen, Inc.
